# Protein Levels of Several Arabidopsis Auxin Response Factors Are Regulated by Multiple Factors and ABA Promotes ARF6 Protein Ubiquitination

**DOI:** 10.3390/ijms21249437

**Published:** 2020-12-11

**Authors:** Keke Li, Sheng Wang, Hong Wu, Hong Wang

**Affiliations:** 1 State Key Laboratory for Conservation and Utilization of Subtropical Agro-Bioresouces, College of Life Sciences, South China Agricultural University, Guangzhou 510642, China; kekeli1013@gmail.com; 2 Department of Biochemistry, Microbiology and Immunology, University of Saskatchewan, Saskatoon, SK S7N 5E5, Canada; sheng.wang@usask.ca

**Keywords:** abscisic acid, Arabidopsis, auxin response factor, ARF6, protein level, protein ubiquitination, temperature stress

## Abstract

The auxin response factor (ARF) transcription factors are a key component in auxin signaling and play diverse functions in plant growth, development, and stress response. ARFs are regulated at the transcript level and posttranslationally by protein modifications. However, relatively little is known regarding the control of ARF protein levels. We expressed five different ARFs with an HA (hemagglutinin) tag and observed that their protein levels under the same promoter varied considerably. Interestingly, their protein levels were affected by several hormonal and environmental conditions, but not by the auxin treatment. ABA (abscisic acid) as well as 4 °C and salt treatments decreased the levels of HA-ARF5, HA-ARF6, and HA-ARF10, but not that of HA-ARF19, while 37 °C treatment increased the levels of the four HA-ARFs, suggesting that the ARF protein levels are regulated by multiple factors. Furthermore, MG132 inhibited the reduction of HA-ARF6 level by ABA and 4 °C treatments, suggesting that these treatments decrease HA-ARF6 level through 26S proteasome-mediated protein degradation. It was also found that ABA treatment drastically increased HA-ARF6 ubiquitination, without strongly affecting the ubiquitination profile of the total proteins. Together, these results reveal another layer of control on ARFs, which could serve to integrate multiple hormonal and environmental signals into the ARF-regulated gene expression.

## 1. Introduction

The auxin signaling cascade from auxin perception to activation of auxin-inducible genes has several core components including an SCF (SKP1–Cullin1–F-box) E3 complex, Aux/IAA and ARF (Auxin Response Factor) transcription factors [1]. The binding of auxin to the receptor from the TIR1/AFB (Transport Inhibitor Response and Auxin F-Box protein) family of F-box proteins stabilizes the interaction between the SCF^TIR1/AFB^ E3 complex and transcription repressor Aux/IAA proteins, triggering the ubiquitination and degradation of Aux/IAAs. The degradation of Aux/IAA proteins frees ARFs which can bind to the auxin response elements in the promoters of auxin inducible genes and activate their expression [1,2]. Thus, auxin functions essentially through ARFs.

In land plants, *ARF* genes have evolved into three conserved A, B, and C classes or subfamilies [3]. The Arabidopsis genome encodes 23 *ARF* genes, but *ARF23* is a truncated pseudogene [4,5]. Based on the reporter activation assays in transfected protoplasts, ARFs in Class A are considered as transcriptional activators and ARFs in Class B as repressors [6,7]. Class C-ARFs are also considered as transcriptional repressors based mainly on their lack of a glutamine-rich middle region which is important for transcriptional activation [8]. In Arabidopsis, five ARFs (ARF5, 6, 7, 8, and 19) are considered as transcription activators while the others as repressors in Class B and C [4,9]. However, evidence for the majority of the repressor ARFs to function as transcription repressors remained to be obtained [5,9]. Since much of the understanding on ARFs has been gained using Arabidopsis as a model, when we describe a specific ARF here it usually refers to the Arabidopsis gene or protein unless a plant species is specified otherwise.

Most ARFs possess three functional domains, an N-terminal DNA binding domain (DBD), a middle region (MR), and a C-terminal PB1 domain [2]. The conserved DBD at the N-terminus is responsible for binding the auxin responsive element [6,10]. The transactivator ARFs in Class A contain a glutamine-rich middle region important for transcriptional activation, which is missing in Class C-ARFs [7,8]. The PB1 domain (formerly referred to as domain III/IV) mediates homo- and hetero-oligomerization with Aux/IAAs and ARFs [11,12,13].

The interactions of ARFs with the Aux/IAA proteins are an important aspect of auxin signaling and its regulation. Since one ARF can interact with multiple Aux/IAAs and vice versa, there is a complex web of interactions among different ARFs and different Aux/IAAs [14]. Results from the yeast two-hybrid studies have shown that the activator ARFs (ARF5, 6, 7, 8, and 19) have more interactions with Aux/IAA proteins compared with the repressor-type ARFs [15,16]. Since the levels of protein expression for various ARFs and Aux/IAAs may differ between the yeast system and plants, and also the tissue specificity is missing in the yeast system, it remains to be seen to what extent the interactions observed in the yeast system reflect the ARF-Aux/IAA interactions in plants. In addition, ARFs can interact with other transcriptional factors. For instance, ARF4 interacts with the transcription factor KAN4 that has an important role in ovule development [17] and ARF8 interacts with a transcription factor named BPEp to limit petal growth [18]. Further, ARF6 is found to interact synergistically with the light/temperature-regulated transcription factor PIF4 and the brassinosteroid-signaling transcription factor BZR1 to regulate large numbers of target genes, while the gibberellin-inactivated repressor RGA blocks their DNA-binding activities [19], revealing more complex interaction among the transcription factors.

Most of the information regarding the functions of *ARF* genes has been obtained using *arf* mutants [4,5,20]. Due to the functional redundancy among the closely related *ARF* genes, the majority of single mutants for 18 Arabidopsis *ARF* genes did not show obvious phenotypical changes [21], although single mutants of *ARF2*, *ARF3*, *ARF5, ARF7*, and *ARF8* displayed phenotypes revealing the functions in several aspects of plant growth and development [22,23,24,25]. For instance, *ARF5* functions in the establishment of vascular and body patterns in embryonic and post-embryonic development [24]. In the *arf7/nph4-1* mutant, the expression of auxin-responsive reporter genes was much reduced in transfected mesophyll protoplasts which could only be partially restored by other *ARF*s, suggesting that *ARF7* has a major role in regulating a subset of genes in mesophyll cells [26]. Results from various studies using double and multiple mutants helped to identify many different functions of *ARF*s particularly for the Class-A *ARF*s (*ARF5* to *8* and *ARF19*) [5,20]. For instance, *ARF6* and *ARF8* have been found to function in adventitious root formation [27], hypocotyl elongation (*ARF6*, *7*, and *8*) [28] and flower organ development and maturation [29,30], while *ARF7* and *ARF19* are involved in the regulation of phosphate starvation response [31]. In addition, *ARF1* and *ARF2* of Class B have a role in regulating senescence and floral organ abscission [32]. ARF2 also functions together with a transcription factor (PLT1) and an auxin transporter PIN1 to control ABA-mediated root meristem activity [33]. Furthermore, *ARF10* of Class C is found to be important for root cap formation [34], in vitro callus initiation and shoot regeneration [35,36]. These results demonstrate that *ARFs* play important and diverse functions in plants.

It is well known that the *ARF* genes are transcriptionally regulated and different *ARFs* have different expression levels and patterns in different tissues [4,37]. In one study, the expression of all 23 *ARF*s in embryogenesis and primary root meristem was characterized using the promoter: GFP reporter approach [38]. The results revealed complex cell-specific patterns and that different *ARF*s had overlapping expression in different cells. Another systematic analysis using in situ hybridization revealed that 13 Arabidopsis *ARF*s were expressed in the shoot apex, showing differences in the pattern and level [15]. One commonality among the expressed *ARF*s is that they tended to be expressed at higher levels in the periphery of the shoot meristem. In addition, *ARF* transcript levels are also regulated by other mechanisms such as alternative transcript splicing and microRNAs [5,37].

The functions of ARFs are also regulated by posttranslational protein modifications. ARF2 can be phosphorylated by a brassinosteroid (BR)-regulated kinase BIN2, leading to the loss of ARF2 DNA binding and repression activities [39]. ARF2 is also phosphorylated under low potassium stress [40]. ARF2 normally represses the expression of a potassium transporter gene *HAK5*, and the phosphorylation abolishes ARF2 binding to *HAK5* promoter and relieves the repression. In addition, BIN2 has been reported to phosphorylate ARF7 and ARF19, and in contrast to reduce activity of ARF2, ARF7, and ARF19 phosphorylation results in the enhanced transactivation activity, which is attributed to reduced ARF7 and ARF19 interactions with the Aux/IAA repressors [41]. On the other hand, ARF7 is modified with the small ubiquitin-like modifier protein and the sumoylation inhibits the interaction with Aux/IAA3 and affects root branching [42].

In terms of the control of protein stability and degradation, there are only a few reports on the control of ARF1 and ARF2 protein levels. Arabidopsis ARF1 is fast degraded in plants and the degradation is inhibited by MG132, a 26S proteasome inhibitor [43]. In addition, ARF1 degradation is not affected in a *CUL1* mutant background and by auxin treatment, suggesting that ARF1 is degraded by the 26S proteasome, but the degradation is through a CUL1-independent pathway [43]. It has been reported that ARF2 protein expression driven by the native promoter is decreased by ethylene, which is inhibited by MG132 [22]. On the other hand, ARF2 protein abundance is enhanced by gibberellin (GA) treatment [44]. Recently, ARF6, ARF8, and ARF17 were also shown to be unstable and their levels were enhanced by MG132 [45]. Apart from these studies, there has been little information on the protein-level control of other ARFs and further regarding how different hormonal and environmental conditions affect ARF protein levels. In this study, we investigated the protein expression of several ARFs and observed that their levels are affected by several different conditions. We further demonstrated that ABA treatment promotes the ubiquitination and degradation of ARF6.

## 2. Results

### 2.1. Analysis of Five ARF Protein Expression in Transgenic Arabidopsis Plants

**Figure 1:**
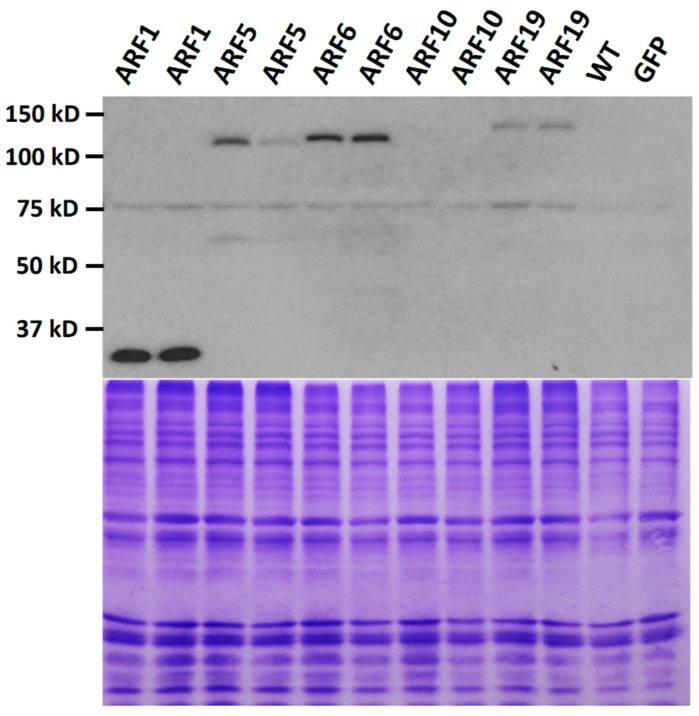
Analysis of HA-ARF protein expression in primary Arabidopsis transformants. Arabidopsis plants were transformed with the HA-tagged *ARF* constructs. Each protein sample was prepared from a pool of 25 non-selected independent transformants, as well as the control plants, to represent an average expression level for the particular construct. For each construct, two replicate samples were prepared. The protein samples were used for electrophoresis and the HA-ARF fusion proteins were detected by western blotting using an anti-HA antibody. Upper panel: western blotting image, with protein molecular weight markers indicated at the left. There is a weak non-specific band present in all samples. Lower panel: image of the gel stained with Coomassie blue to show the amount of samples loaded.

To study protein expression, we prepared the plant expression constructs of several ARFs tagged with the HA (hemagglutinin) epitope. We selected three activator *ARF*s from Class A, *ARF5* (At1g19850), *ARF6* (At1g30330), and *ARF19* (At1g19220), due to the importance of the activator ARFs in activating auxin-regulated genes, as well as *ARF1* (At1g59750) from Class B and *ARF10* (At2g28350) from Class C [9] for the study. We used the widely used 35S promoter which is not known to be significantly regulated by auxin or any other hormones, so that we could separate the protein control from the transcriptional control by the native *ARF* promoters. First, Arabidopsis primary transformants of each construct were analyzed for HA-ARF protein expression by western blotting. Each protein sample was prepared from a pool of independent Arabidopsis transformants (about 25 in each plate) to represent the average level of the HA-ARF protein for these transformants. With the expected molecular size of the 2× HA tag (3.0 kD) included, the theoretical sizes of HA-ARF1, HA-ARF5, HA-ARF6, HA-ARF10, and HA-ARF19 are 76.7, 102.6, 106.3, 79.7, and 123.6 kD, respectively. As shown in Figure 1, no HA-specific band was detected in the wild type and transgenic control of a GFP (green fluorescence protein) line. A full-length HA-ARF protein band was detected in HA-ARF5, HA-ARF6, and HA-ARF19 transformants, while only a strong partial HA protein band was detected in ARF1 transformants and no band was detected in ARF10 transformants. Also, it was noted that the two replicate samples were similar to each other for *HA-ARF* constructs, indicating that the protein samples prepared from a pool of 25 independent transformants effectively represent the average expression level of the construct.

The purpose of the initial analysis was to determine and compare the protein expression among the five ARFs, using pools of 25 primary transformants. However, the protein level could vary greatly among different transformants. Thus, individual T1 lines were also analyzed. Among at least 12 lines screened for each construct, T1 lines with a full-length HA-ARF protein band was identified for HA-ARF5, HA-ARF6, and HA-ARF19 while lines with only partial band were identified for HA-ARF1 (Figure S1). There were more T1 lines (7 out of 12 lines screened) with the full-length protein for HA-ARF6, consistent with the strongest protein expression level in the primary transformations on average. The initial analysis of HA-ARF10 lines did not identify a line with the full-length protein. Using a commercial gel system and different conditions, three T1 lines with a full-length protein band were identified (Figure S1). These results collectively indicate that (1) the protein expression level varies greatly among different ARFs when they are driven by the same 35S promoter, with ARF6 having the strongest expression level among the five ARFs analyzed, and (2) ARF1 is easily degraded resulting in only a strong partial HA-ARF1 band detected in some lines. Stable and homozygous transgenic lines of subsequent generations were selected and used in further analysis.

### 2.2. Effects of Hormonal and Environmental Treatments on ARF Protein Expression

**Figure 2:**
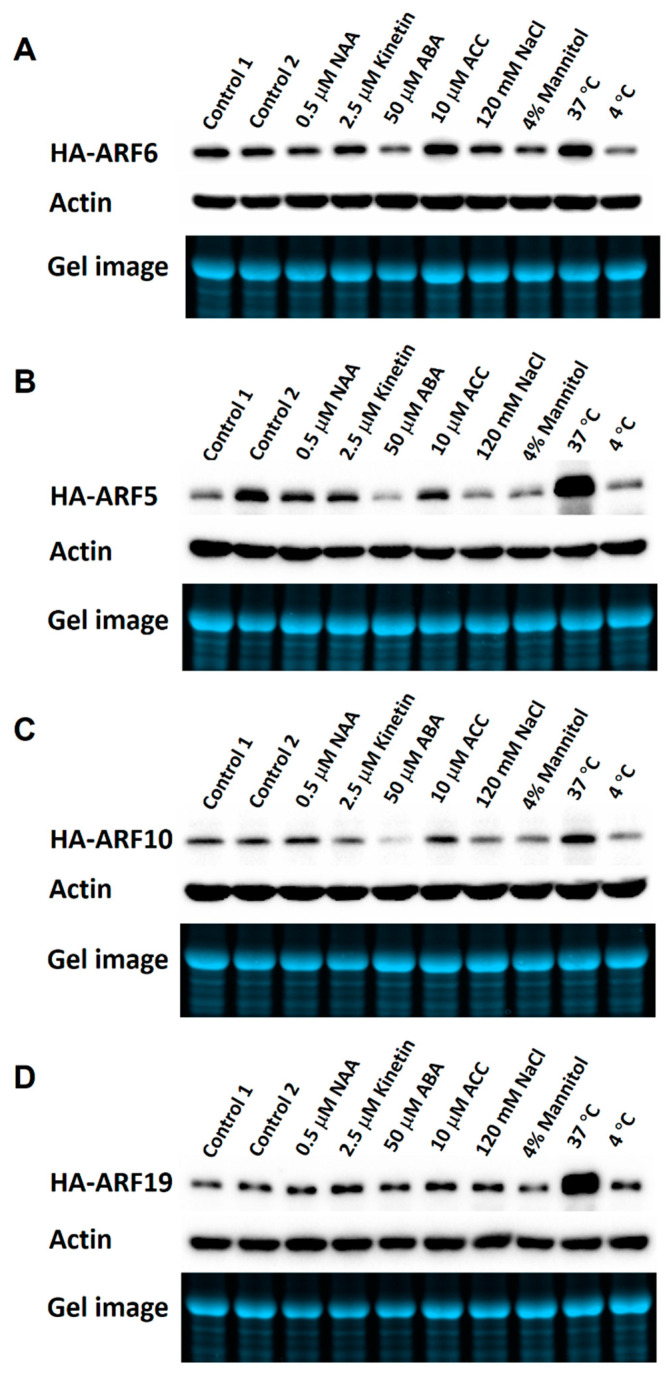
Effects of different treatments on protein expression of HA-ARF6 (**A**), HA-ARF5 (**B**), HA-ARF10 (**C**), and HA-ARF19 (**D**). Stable transgenic *HA-ARF* lines (T2 or later generation) were used. Seedlings were grown vertically in Petri plates. At 10 to 11-day stage they were transferred to treatment plates containing a piece of filter paper and 5 mL half-strength MS with or without a treatment reagent, with each plate having about 25 seedlings. After the treatment, protein samples were prepared and used (30 μg per lane) for electrophoresis in the Bio-Rad TGX mini-gel, followed by western blotting. In each subfigure, the first row shows the western blot with an anti-HA antibody. For loading controls, the second row shows the western blot with an anti-actin antibody and third row shows the gel image. The treatment conditions are indicated above the lanes. Control 1: half MS medium; Control 2: half MS medium plus 0.1% ethanol (since NAA, kinetin, ABA were added from ethanol stocks); ACC: 1-aminocyclopropane-1-carboxylic acid.

To determine the influence of plant hormones and environmental conditions on ARF protein expression, various hormonal and stress treatments were applied to Arabidopsis seedlings of *HA-ARF* transgenic lines. After 8 or 12 h of treatment, protein samples were prepared from treated seedlings and used in western blotting analysis. For HA-ARF6, ABA (abscisic acid) and 4 °C treatments clearly reduced the protein level in the treated seedlings compared to the controls, while 37 °C and interestingly 1-aminocyclopropane-1-carboxylic acid (ACC, an ethylene precursor) treatments increased HA-ARF6 level (Figure 2A). The results for HA-ARF5 (Figure 2B) and HA-ARF10 (Figure 2C) are similar. ABA treatment reduced their levels while 37 °C treatment increased them. Also, 4 °C, NaCl and mannitol treatments reduced the levels slightly (Figure 2B,C). For HA-ARF19, the protein level remained unchanged for all treatments except for a strong increase by 37 °C treatment (Figure 2D). Considering all four ARFs together, they share the following similarities: a strong increase by 37 °C treatment for all four ARFs, a clear decrease by ABA for all but HA-ARF19, and a milder decrease by 4 °C treatment, NaCl and mannitol treatments for all but HA-ARF19. The increase by ACC treatment seems specific for HA-ARF6. It is interesting to note that NAA (1-naphthaleneacetic acid) treatment did not affect the protein level of any of the ARFs. There results show that several hormonal and environmental factors instead of auxin can affect ARF protein levels and further their effects may vary among different ARFs.

**Figure 3:**
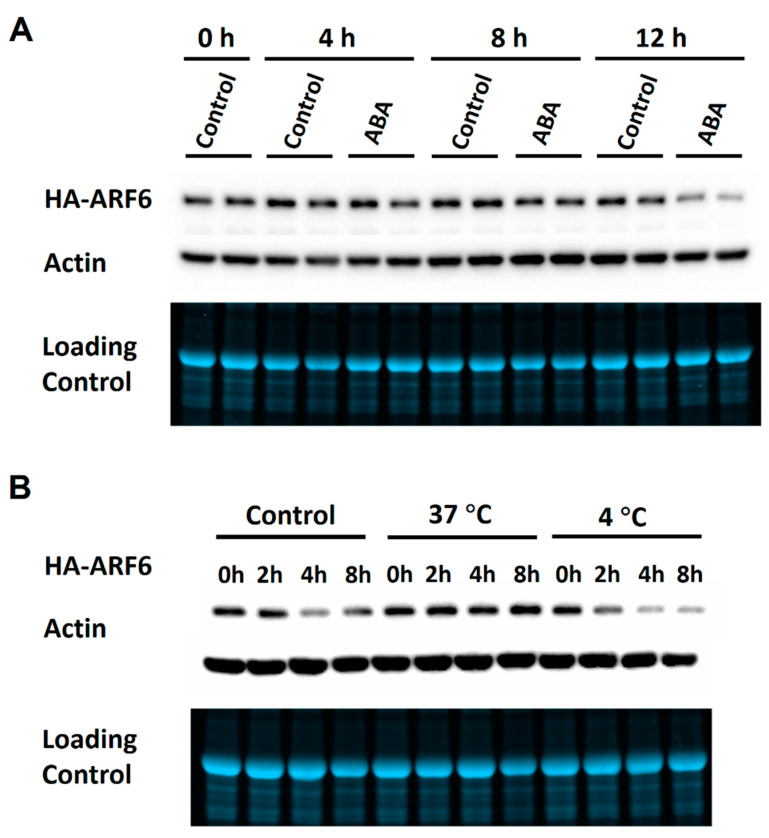
Time-course analysis of ABA and temperature treatments on HA-ARF6 protein levels. Transgenic *HA-ARF6* seedlings were grown vertically in Petri plates. For ABA treatments, seedlings (10 to 11-day old) were transferred to treatment plates containing a piece of filter paper and 5 mL half-strength MS with or without a treatment reagent, with each plate having about 25 seedlings. For temperature treatments, seedlings grown on one-half MS plates were treated directly at the temperatures with the control at 22 °C. After the treatments, protein samples were used for electrophoresis (30 μg per lane) in the Bio-Rad TGX mini-gel, followed by western analysis. In each subfigure, the first row shows the western blot with an anti-HA antibody, the second row shows the western blot with an anti-actin antibody and the third row shows the gel image. The treatment conditions are indicated directly above the lanes. (**A**) ABA treatments. Seedlings were treated in control (one-half MS medium plus 0.1% ethanol) and 50 μM (+)-ABA for the indicated hours. Each time point had two replicate samples. (**B**) Temperature treatments. Seedlings were treated for the indicated hours with the control at 22 °C.

**Figure 4:**
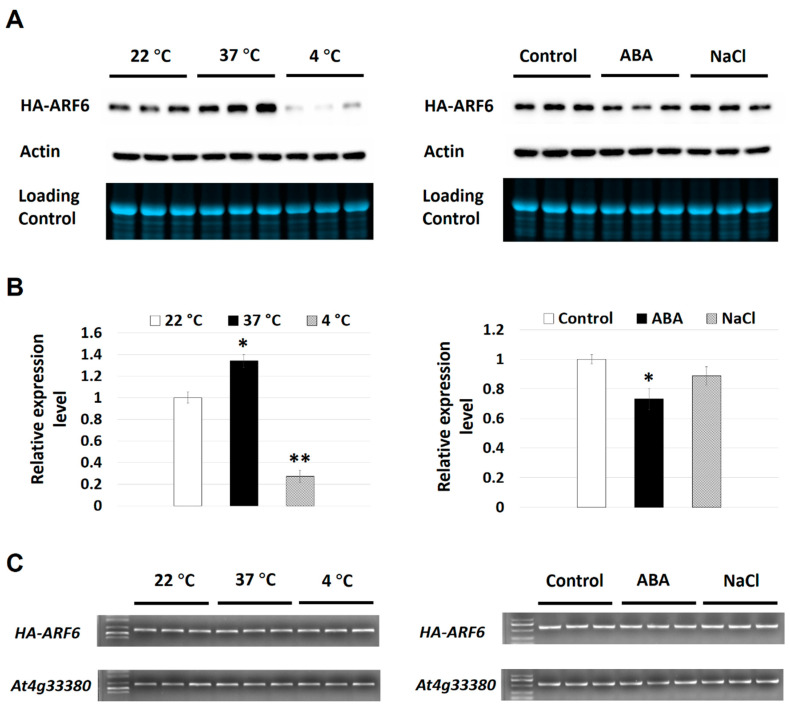
Effects of temperature, ABA, and NaCl treatments on HA-ARF6 expression. Transgenic *HA-ARF6* seedlings were grown vertically in Petri plates. Seedlings (10-day old) were treated directly (for temperature treatments) or transferred to new plates containing a filter paper and 5 mL half-strength MS with or without the treatment reagent. Each plate had about 25 seedlings. After 8 h of treatment, protein and RNA samples were prepared. (**A**) Effects of temperature, ABA, and NaCl treatments by western analysis (30 μg protein per lane). The first row shows the western blot with an anti-HA antibody. For loading controls, the second row shows the western blot with an anti-actin antibody and third row shows the gel image. The treatment conditions are indicated above the lanes. Left part: temperature treatments. Right part: (+)-ABA (50 μM) and NaCl (120 mM) treatments. The control had half MS medium plus 0.1% ethanol. (**B**) Quantitative analysis of the western blots shown in (**A**). The intensities of HA-ARF6 bands were measured using Bio-Rad Image software 15.2.1. Student’s *t*-test was performed to determine the difference between the treatment and control. For each treatment, the average of three replicates is shown with the standard error. The sign above the bar indicates a significant difference from the control: * *p* < 0.05, ** *p* < 0.01. (**C**) Transcript analysis. RNA samples were isolated from treated seedlings with each treatment having three replicates. cDNAs were synthesized and used in RT-PCR analysis. *HA-ARF6* was amplified using a primer specific to the HA sequence and reverse primer for *ARF6*. The gene *At4g33380* was used as a load control.

To further verify the observations, time-course experiments were performed for ABA, 37 °C and 4 °C treatments on the expression of HA-ARF6. The ABA treatment resulted in a slight reduction in the HA-ARF6 level after 8 h of treatment and a clear reduction after 12 h of treatment compared to the control (Figure 3A). For the temperature treatments, seedlings at 37 °C had a higher level of HA-ARF6 and those at 4 °C had a lower level of HA-ARF6 after 4 or 8 h of treatment, compared to the controls (Figure 3B). We further analyzed the treatment effects quantitatively with each treatment having 3–5 replicates. The results showed that HA-ARF6 protein was significantly reduced by 4 °C, and ABA treatments, but significantly increased by 37 °C treatment (Figure 4A,B). The 120 mM NaCl treatment resulted in a reduction in the HA-ARF6 level, although the reduction was not statistically significant based on the quantitative data. To determine whether the changes in the HA-ARF6 level caused by different treatments were due to a change at the transcript level, RT (reverse transcription)-PCR was performed to detect the *HA-ARF6* transcripts using an HA-specific primer and an *ARF6*-specific reverse primer. The results showed that the *HA-ARF6* transcript level remained consistent and was not affected by these treatments (Figure 4C). Furthermore, we determined the transcript level by quantitative real-time PCR (qPCR). The results indicated that *HA-ARF6* transcript level did not change much when Arabidopsis seedlings were treated under 37 °C, ABA and NaCl conditions, but showed a slight increase at 4 °C compared to the control (Figure S2). Further examination of the qPCR data (Table S1) revealed that the level of the control gene *At4g33380* showed a decrease under 4 °C treatment and normalization of *HA-ARF6* level using *At4g33380* resulted in an increase in the *HA-ARF6* level. Together, the RT-PCR and qPCR results indicated that *HA-ARF6* transcript level did not change much under different treatments and in particular it was not increased by the 37 °C treatment or decreased by ABA treatment, suggesting that the observed changes in the *HA-ARF6* protein level by the treatments are not due to a change in the transcript level, but due to posttranscriptional control on the protein level.

**Figure 5:**
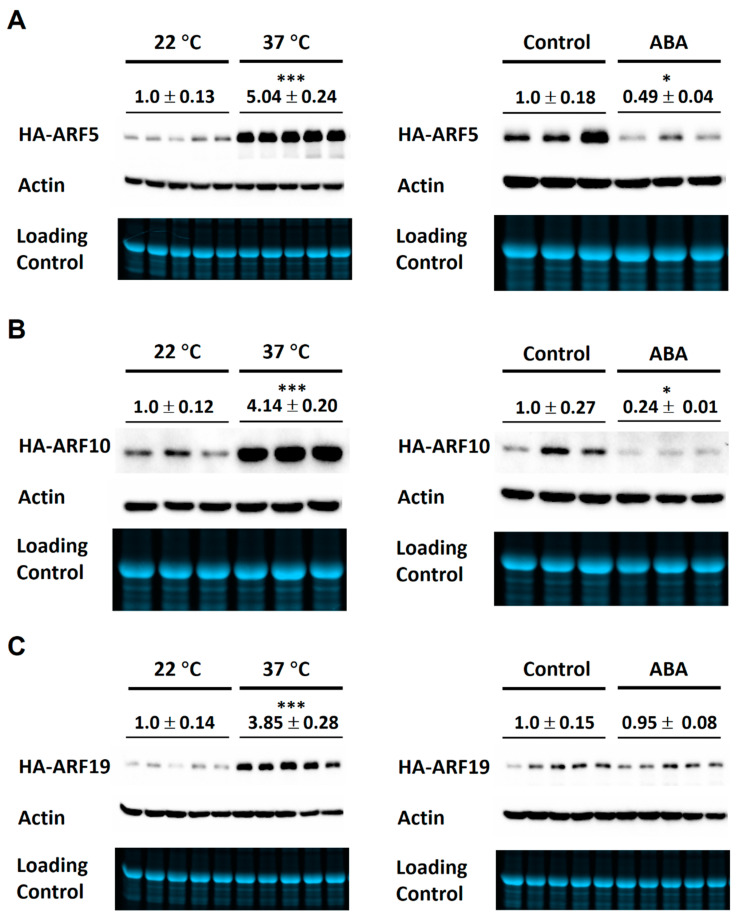
Effects of high temperature and ABA on the expression of ARF5, ARF10, and ARF19. Transgenic seedlings of *HA-ARF6*, *HA-ARF10*, and *HA-ARF19* were grown vertically in Petri plates. For temperature treatments, seedlings (10-day old) in the plates were treated directly at the indicated temperatures for 8 h. For ABA treatments, seedlings were transferred to new plates containing a filter paper and 5 mL half-strength MS with or without 50 μM (+)-ABA. Each plate had about 25 seedlings. After the treatment, protein samples were used (30 μg per lane) in electrophoresis and western blotting. In each subfigure, the first row shows the western blot with an anti-HA antibody, the second row shows the western blot with an anti-actin antibody and third row shows gel image. The treatment conditions are indicated above the lanes, and relative level of *ARF* protein is shown below the treatment, based on three or five replicates. (**A**) HA-ARF5. (**B**) HA-ARF10. (**C**) HA-ARF19. The controls for ABA treatments used half MS medium plus 0.1% ethanol. Student’s *t*-test was used to determine the difference between the treatment and control. The sign above the average value indicates a significant difference from the control: * *p* < 0.05, *** *p* < 0.001.

We then examined the effects of ABA and 37 °C treatments on the protein levels of HA-ARF5, HA-ARF10, and HA-ARF19. As shown in Figure 5, 37 °C treatment of 8 h strongly increased the levels of HA-ARF5, HA-ARF10, and HA-ARF19 by similar 3–4 folds. The 50 μM ABA treatment decreased the levels of HA-ARF5 and HA-ARF10, but not that of HA-ARF19 (Figure 5). Statistical analysis revealed that the changes by ABA treatments were significant for three of the four ARFs except for HA-ARF19. Taken together, those results show clearly that the 37 °C treatment strongly increases the ARF protein levels, while ABA treatment reduces the levels of HA-ARF5, HA-ARF6, and HA-ARF10, but not HA-ARF19, consistent with earlier observations (Figure 2).

### 2.3. Effects of ABA and Temperature Treatments on the Degradation and Ubiquitination of HA-ARF6

**Figure 6:**
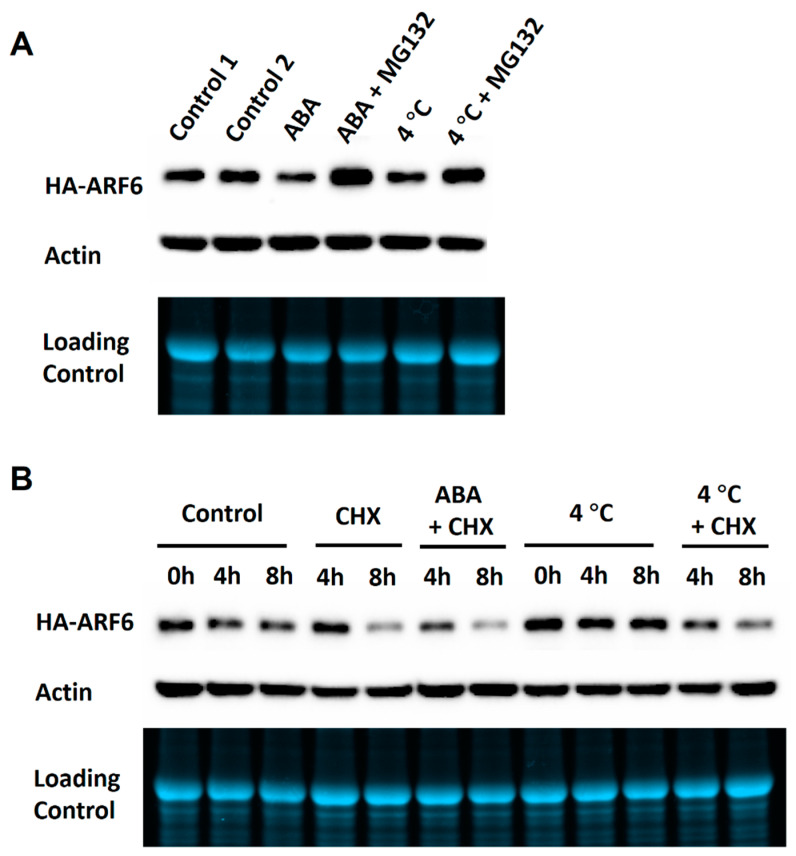
Effects of ABA and 4 °C treatments on HA-ARF6 level in the presence of MG132 or cycloheximide (CHX). Transgenic *HA-ARF6* seedlings were grown vertically in Petri plates. Seedlings (10-day old) were transferred to new plates containing a filter paper and 5 mL half-strength MS with or without the treatment reagent. Each plate had about 25 seedlings. After 4 or 8 h of treatment, proteins were extracted and used in electrophoresis (30 μg protein per lane) in Bio-Rad TGX mini-gels and western blotting. In each subfigure, the first row shows the western blot with an anti-HA antibody. For loading controls, the second row shows the western blot with an anti-actin antibody and third row shows the gel image. The treatment conditions are indicated above the lanes. (**A**) ABA and 4 °C treatments with or without MG132. Control 1: normal half MS medium; Control 2: half MS medium plus 0.1% ethanol and 0.1% DMSO (ABA and MG132 stocks were prepared in ethanol and DMSO, respectively); ABA: 50 μM; MG132: 25 μM. (**B**) ABA and 4 °C treatments with or without CHX. Control: half MS medium plus 0.1% ethanol and 0.1% DMSO; ABA: 50 μM; CHX: 200 μM.

To determine whether the reductions of the HA-ARF6 level by ABA and 4 °C treatments were through protein degradation, we treated the HA-ARF6 seedlings with or without MG132, an inhibitor of the 26S proteasome. Confirming earlier results, ABA and 4 °C treatments decreased HA-ARF6 level. On the other hand, the seedlings treated with ABA or under 4 °C in the presence of MG132 had a higher HA-ARF6 level than that of the control (Figure 6A and Figure S3A), suggesting that ARF6 is degraded through 26S proteasome. We further performed the ABA and 4 °C treatments with or without cycloheximide (CHX), an inhibitor of eukaryotic protein synthesis. The seedlings treated with ABA or under 4 °C in the presence of CHX had a lower HA-ARF6 level compared to the treated seedlings without CHX (Figure 6B and Figure S3B), indicating that HA-ARF6 was depleted faster when protein synthesis was inhibited. The observations that HA-ARF6 level was reduced under ABA and 4 °C treatments, but was increased in the presence of MG132 suggest that ABA and 4 °C treatments reduce the HA-ARF6 level by increasing protein degradation through the 26S proteasome.

**Figure 7:**
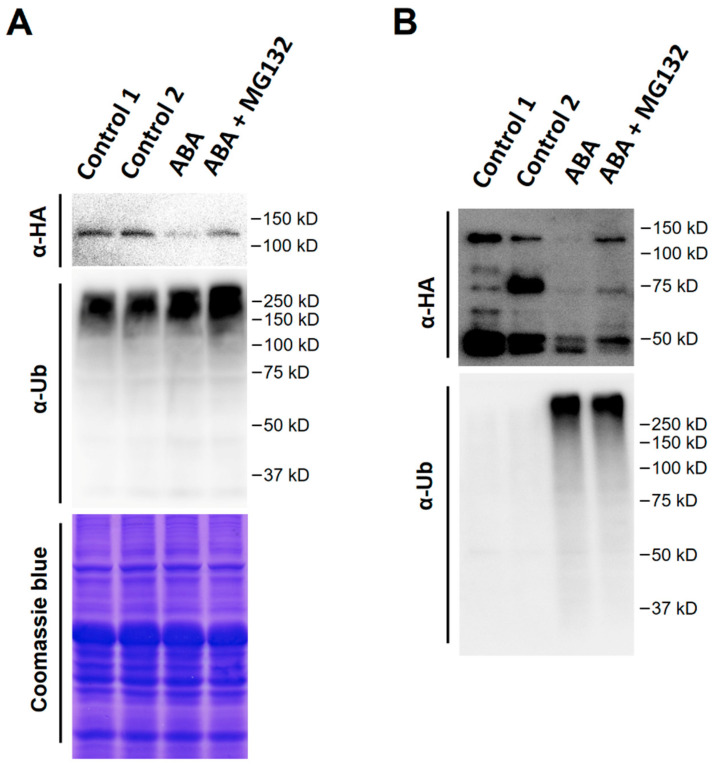
Effect of ABA treatment on the ubiquitination of HA-ARF6. Transgenic *HA-ARF6* seedlings were grown vertically in Petri plates. Seedlings (10-day old) were transferred to plates containing a filter paper and 5 mL half-strength MS with or without the treatment reagent. Each plate had about 25 seedlings. After 8 h of treatment, protein samples were prepared. (**A**) Western blotting using total protein samples (30 μg protein per lane). The top panel shows the western blot with an anti-HA antibody, the second panel shows the western blot with an anti-Ub antibody, and the bottom panel shows gel staining with Coomassie blue. The treatment conditions are indicated directly above the lanes. Control 1: normal half MS medium; Control 2: half MS medium plus 0.1% ethanol and 0.1% DMSO; ABA: 50 μM; MG132: 25 μM. (**B**) Analysis of purified HA-ARF6. Total proteins (600 μg for each sample) were used to purify HA-ARF6 with anti-HA agarose beads. The purified HA-ARF6 samples were used in western blotting. The top panel shows the western blot with an anti-HA antibody and bottom panel shows the western blot with an anti-Ub antibody.

These findings led us to ask whether ABA treatment increases HA-ARF6 protein ubiquitination. To address the question, ABA and control treatments were applied to the seedlings with or without MG132. Total protein samples were prepared from treated and control HA-ARF6 seedlings. Also, the HA-ARF6 protein was purified by immunoprecipitation using anti-HA-conjugated agarose beads and eluted with HA peptide. The total and purified HA-ARF6 proteins were analyzed by western blotting, with an anti-HA antibody for HA-ARF6 protein and an anti-Ub antibody for the ubiquitinated proteins. For the total protein samples, ABA treatment reduced HA-ARF6 level compared to the control while MG132 increased its level (Figure 7A). The ubiquitinated protein profiles of the total proteins for the ABA and ABA + MG132 treatments were comparable to the two controls, although the ABA + MG132 treatment appeared to have slightly increased amount of protein ubiquitination (Figure 7A, mid-panel, showing a slightly stronger and longer profile). For the purified HA-ARF6 protein samples, ABA treatment sample had a decreased amount of the full-length HA-ARF6 level and the decrease was inhibited by MG132 (Figure 7B). Western blotting of the purified HA-ARF6 samples with the anti-Ub antibody revealed a drastic increase of ubiquitinated proteins in both the ABA and ABA + MG132 samples compared to the controls, suggesting that ABA treatment strongly promotes HA-ARF6 ubiquitination. Furthermore, the band intensity increased with the increasing molecular sizes, suggesting that higher molecular weight proteins with stronger signal may be due to HA-ARF6 protein having increasing number of Ub moieties (longer poly Ub-chain). It is also interesting to note that although the ABA + MG132 treatment had a higher level of HA-ARF6 than the ABA treatment, the profiles of ubiquitinated proteins for both treatments were similar (Figure 7B, bottom panel), suggesting that although MG132 increased HA-ARF6 level by inhibiting the 26S proteasome activity, it did not significantly affect the abundance of ubiquitinated HA-ARF6 protein under ABA treatment.

## 3. Discussion

### 3.1. Different ARFs Have Very Different Expression Levels under the Control of the Same Promoter

Since auxin functions essentially through ARFs and ARFs play important roles in many and diverse plant growth and developmental processes, it is critical to understand how ARFs are regulated. Extensive results have shown that *ARF* genes are regulated at transcriptional levels and in addition ARF proteins can be modulated through protein modifications such as phosphorylation (see Introduction). However, little is known regarding the control of ARF protein levels. In this study, we investigated ARF protein regulation and how they may be affected by different factors. Furthermore, we used ARF5, ARF6, and ARF19 from Class A, which are the transcription activators, as well as ARF1 and ARF10 which represents Class B- and Class C-ARFs, respectively [9]. The ARFs were tagged with a short HA tag so that the recombinant HA-ARF proteins could be detected conveniently and specifically.

The expression levels of HA-ARF proteins were generally low, even though the strong 35S promoter was used. When groups of primary transformants were analyzed, only HA-ARF5 and HA-ARF6 had a clear band while HA-ARF1 and HA-ARF10 were not detected (Figure 1). We had to use a large amount of protein sample to identify T1 transgenic lines expressing the HA-ARF protein (Figure S1). Also, for HA-ARF1, no full-length protein was detected but a strong partial band was observed suggesting that HA-ARF1 was fast degraded. Similarly, it has been shown that Arabidopsis ARF1 is fast degraded in plants and the degradation is inhibited by MG132 [43]. Results from a recent study also indicate that ARF6, ARF8, and ARF17 are unstable proteins [45]. Together, these observations suggest that ARF proteins in general are relatively unstable in plants.

We observed that the protein levels of the five ARFs varied and the three activator ARFs generally had a higher level than the two repressor ARFs. Since the protein samples from the primary transformants were prepared from groups of 25 independent transformants, the HA-ARF level in one protein sample should represent the average expression level of a particular *HA-ARF* construct, which is supported by the consistency between the two duplicate samples (Figure 1). Since one important molecular property of ARFs is the formation of hetero- and homodimers with Aux/IAA and ARF proteins, protein levels or concentrations in cells are expected to impact the those interactions. Much understanding on ARF and Aux/IAA interactions has been gained using the yeast two-hybrid system in which ARFs are expressed with a yeast promoter. It has been observed that Class A-ARFs interact with more Aux/IAAs than ARFs in Class B and C [15,16]. One interpretation is that those ARFs (ARF5 to 8 and ARF19) are able to interact with more Aux/IAA proteins. Alternatively, considering the present findings, the ARFs with fewer interacting Aux/IAA proteins could also be due to a lower protein level.

### 3.2. Protein Levels of Four ARFs Are Modulated Posttranslationally by Multiple Factors

We observed that none of the HA-ARF5, HA-ARF6, HA-ARF10, and HA-ARF19 protein levels under the control of 35S promoter was affected by NAA treatment, consistent with previous studies showing that the protein levels of ARF1, ARF2, and ARF7 were not affected by auxin treatments [22,43,44]. The strong increases of the four HA-ARFs by 37 °C treatment and clear reductions of HA-ARF5, HA-ARF6, and HA-ARF10 by ABA treatment (Figure 2 and Figure 5) suggest that other factors can modulate ARF protein levels independent of auxin. Since the 35S promoter is not known to be significantly regulated by plant hormones and temperature, and further we showed clearly that the *HA-ARF6* transcript level was not affected by the temperature, ABA, and NaCl treatments, our results indicate that ABA, 4 °C and 37 °C treatments modulate the ARF protein levels post-translationally. In addition, the 37 °C treatment seems to have a stronger effect on HA-ARF5, HA-ARF10, and HA-ARF19 than on HA-ARF6 based on the fold of change (Figure 4 and Figure 5), while ABA treatment had a clear effect on the ARFs except for HA-ARF19, indicating that these factors can affect ARFs differently.

In addition to the clear effects by ABA and temperature treatments, our results also show that HA-ARF5, HA-ARF6, and HA-ARF10 protein levels were weakly reduced by NaCl and mannitol treatments (Figure 2). Since it is known that the ABA level increases under stress conditions [46], it is conceivable that NaCl and osmotic treatments may decrease the ARF protein levels through ABA. Also, the HA-ARF6 level appeared to be promoted by ACC treatment (Figure 2). There has been no report regarding ethylene promoting the protein level of an activator (Class A) ARF. On the other hand, ethylene was found to decrease ARF2 protein level and this effect was inhibited by MG132, suggesting that ethylene promotes ARF2 degradation through the 26S proteasome [22]. In addition, it is known that auxin and ethylene have complex interactions, acting synergistically in some processes and antagonistically in others [47]. Thus, it is possible that ethylene may affect the stability and degradation of certain ARFs. Collectively, our results have revealed several factors including ABA, temperature (both high and low temperatures) and salt stress can modulate ARF protein levels and their effects may differ among different ARFs. It will be interesting to investigate how various factors may affect the protein levels of other ARFs.

### 3.3. ABA Treatment Promotes the Ubiquitination and Degradation of ARF6

There are complex cross-talks among different plant hormones, which are important for plant development and stress response [47,48,49]. It is thus critical to understand the specific mechanisms that underlie hormone cross-talks. There is some strong genetic evidence on the auxin and ABA interactions. From auxin to ABA direction, it has been shown that the arf10 arf16 double mutant had decreased seed dormancy and lower transcript level of ABI3, an important component in ABA signaling, suggesting that ARF10 and ARF16 may enhance ABA-mediated seed dormancy through the activation of ABI3 [50]. Conversely, ABI3 represses a microRNA (MIR160B) that targets ARF10 and ARF16 [51]. Furthermore, Arabidopsis ARF2 may affect seed size and drought tolerance through regulating ABA signaling [52]. In addition, transgenic expression of a sweet potato *IbARF5* in Arabidopsis and downregulation of a tomato *SlARF4* have been found to increase ABA content in the plants [53,54]. From ABA to auxin direction, an ABI3-like factor from the bean is known to bind an auxin-inducible promoter [55]. Additionally, Arabidopsis *ARF2* expression is induced by ABA treatment and the *ARF2* mutants showed enhanced ABA sensitivity in seed germination and primary root growth [56]. Results from those studies indicate that auxin and ABA interact mostly through gene expression and transcript regulation. Our findings that ABA promotes the degradation of several Arabidopsis ARFs and strongly induces the ubiquitination of HA-ARF6. These results indicate that ABA can regulate ARF protein level by promoting their ubiquitination, thus providing a novel link for the ABA and auxin cross-talk.

Also, it is known that cold stress can change auxin transporters and gradient, thus affecting plant growth [57]. Our observation that the 4 °C treatment promotes ARF6 degradation provides another possible mechanism by which cold stress can affect plant growth. Furthermore, the 37 °C treatment strongly increased the levels of all four ARFs, while ABA did not have an effect on ARF19. Such differences imply that 37 °C treatment affects ARFs through a different mechanism from that of ABA. It is interesting to note that NAA treatment did not affect the level of any of the four ARFs analyzed, suggesting that ABA and temperature treatments affect ARF protein levels likely through auxin-independent pathways. It will be important to identify the specific E2 and E3 components responsible for ARF6 ubiquitination and degradation induced by ABA treatment, and investigate the mechanism by which 37 °C treatment increases ARF protein levels.

### 3.4. ARF Protein-Level Control Provides Another Layer of Regulation for ARFs to Integrate Multiple Hormonal and Environmental Signals

**Figure 8:**
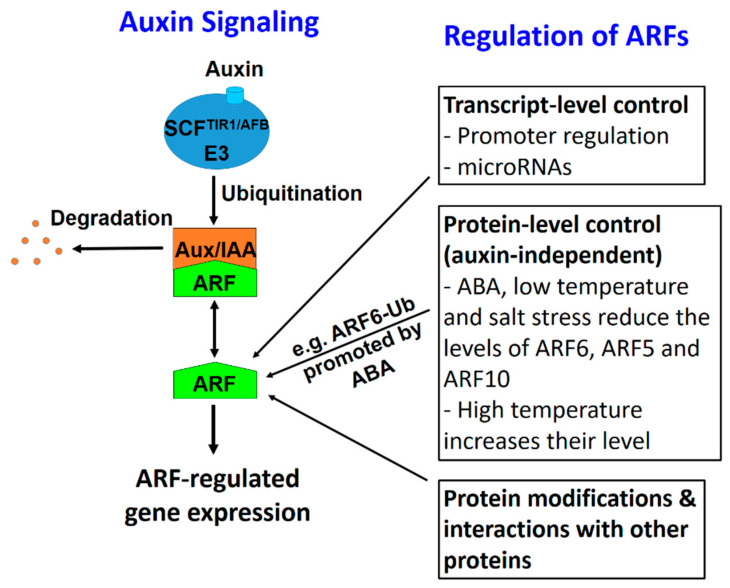
A schematic diagram to show different types of regulation on ARFs, which can integrate multiple signals into the auxin signaling cascade. In auxin signaling, the binding of auxin stabilizes the interaction between the SCF^TIR1/AFB^ E3 complex, resulting in the ubiquitination and degradation of Aux/IAA proteins, which frees ARFs. The ARFs function to activate or suppress ARF-regulated genes. ARFs can be regulated at different levels. Results from this study reveal particularly that several factors can regulate ARF protein levels, likely through auxin-independent mechanisms. ABA, low temperature, and salt stress reduce the levels of certain ARFs while 37 °C temperature increases their levels. Furthermore, ABA promotes ARF6 ubiquitination.

Based on the SCF-Aux/IAA-ARF signaling cascade, auxin functions essentially through ARFs. Experimental evidence so far has shown that ARFs are regulated at several levels (Figure 8). At the transcript level, ARFs are differentially expressed in different cells and tissues (see Introduction). The expression of some ARFs in different plants is regulated by auxin [5]. In addition, the expression of certain *ARF*s may be regulated by other factors. It has been shown that Arabidopsis *ARF19* expression is induced by ethylene [58] and in the root meristem *ARF19* is regulated by a cytokinin-dependent transcriptional factor ARR12 [59]. Additional layers of regulation on ARFs include protein modifications and interactions with other proteins [37]. Results in this study reveal another layer of regulation at the protein level, with some factors reducing the protein levels through increased ARF degradation while other factors enhancing ARF levels (Figure 8). Thus, different hormonal and environmental conditions can converge on ARFs through modulating their protein levels and thus affect ARF-regulated gene expression. These results provide strong support for the notion that ARFs may function to integrate multiple signals in addition to their essential role in auxin signaling.

## 4. Materials and Methods

### 4.1. Plant Growth, Construct Preparation, and Plant Transformation

For normal plant growth and seed harvest, *Arabidopsis thaliana* ecotype “Columbia-0” WT and transgenic lines were grown in a growth room or chamber (20 °C constant, 16/8 h day/night photoperiod with a fluence rate of 90–120 µmoles/m^2^/min).

For preparing *HA-ARF* expression constructs, the *ARF* coding regions were amplified by PCR from Arabidopsis cDNA using *Pfu* DNA polymerase and gene-specific primers listed in Table S2. The DNA fragments were purified and digested with restriction enzymes, before they were cloned into a vector (based on pBI121) containing a 2× HA-tag sequence at the N-terminus [60] to make the *HA-ARF* fusion. The *HA-ARF* fragments were then cloned into a plant expression vector modified from pCambia1300 (http://www.cambia.org/daisy/cambia/585.html) behind the CaMV (cauliflower mosaic virus) 35S promoter.

The constructs were used to transform WT Arabidopsis plants, using the infiltration method [61] with modifications [62]. Seeds from *Agrobacterium*-infiltrated plants were plated on agar plates containing hygromycin (30 mg/L). Hygromycin-resistant transformants were transferred to soil and genotyped using a primer specific to the HA sequence (CCCATACGATGTTCCAGAC) and a primer specific to the *ARF* gene (Table S2). Transgenic lines expressing the HA-ARF protein were further confirmed by western blotting (see below). At least two independent lines were analyzed initially. For most of the analysis, homozygous stable transgenic lines of T2 or later generations were used.

### 4.2. Seedling Growth and Treatments

Seeds of transgenic *HA-ARF* and control Arabidopsis lines were sterilized, stored at 4 °C for two days and then plated on one-half MS agar plates (100 × 15 mm) containing 0.5% sucrose and 0.7% agar. Plates were placed vertically in a plant tissue culture chamber (20 °C constant, 16/8 h day/night photoperiod). Seedlings of 10–11 day old were used in general for various treatments.

For temperature treatments, plates with the seedlings were placed at the indicated temperatures. For other treatments, seedlings were transferred to treatment plates containing a piece of filter paper and 5 mL half-strength MS with or without a treatment reagent. The treatment plates with the plate lid on were placed in a box on a rotary platform with slow rotation. The treatment conditions used are: NAA (auxin), kinetin (cytokinin), (+)-ABA, ACC (1-aminocyclopropane-1-carboxylic acid), NaCl, mannitol, and temperature (22 °C, 4 °C, and 37 °C) treatments. The concentrations for these reagents were selected based on previous testing done in our laboratory and they were specified in each figure. Following the treatment, about 25 seedlings from one plate were used to prepare one protein sample.

### 4.3. Protein Extraction, Western Blotting, and Quantitative Analysis

Plant tissues (seedlings) were homogenized with a plastic pestle in a 1.5 mL tube containing the protein extraction buffer (50 mM Tris-HCl pH 8.0, 200 mM NaCl, 10 mM DTT (dithiothreitol), 1% (v/v) Triton X-100, Sigma protease inhibitor cocktail (Sigma-Aldrich no. P9599, Oakville, ON, Canada)). Following centrifugation at 14,000× *g* for 10 min at 4 °C, the supernatant was transferred into a fresh tube and centrifuged again for 5 min. Bradford protein assay [63] was used (with Bradford reagent from Bio-Rad) to determine the total protein concentration, and the protein samples were stored at −80 °C for further analysis.

Gel electrophoresis was performed in general as described [64] with a few modifications. Majority of analysis used min-gels (10% resolving gels) prepared from the TGX Stain-Free FastCast Acrylamide Kit (Bio-Rad, no. 1610183, Hercules, CA, USA) and gel images were obtained using the Bio-Rad Gel DocXR+ imaging system. Some gels were prepared using regular acrylamide solution (29:1 mix). Following electrophoresis, proteins were transferred onto PVDF membrane (Bio-Rad) using a Bio-Rad Trans-Blot apparatus at a constant voltage of 25 V for 12 h in 4 °C.

Western blotting was performed as described [64]. For detecting HA-ARF fusion proteins, mouse monoclonal anti-HA antibodies (YEASEN no. 30701ES20, Beijing, China or Santa Cruz no. sc-7392, Dallas, TX, USA) were used. The loading control used a mouse monoclonal anti-actin antibody (Abbkine no. A01050, Wuhan, China; 1:5000). For quantitative analysis, the intensity of each protein band was measured using Bio-Rad Image lab software 15.2.1. The relative band intensity was obtained with the average of the control treatment set at 1.0. Student’s *t*-test (two-tail, unequal varience) was used to analyze the difference between the control and treatment values.

### 4.4. HA-ARF Protein Pulldown by Immunoprecipitation

For HA-ARF6 protein pulldown, total protein extract (600 μg) was incubated with anti-HA agarose beads (Sigma-Aldrich, Oakville, ON, Canada) at 4 ℃ for 1 h (on a tumbler). The beads were washed twice with the wash buffer (10 mM Tris-HCl at pH 8.0, 5 mM NaCl, 0.05% Tween, 0.5 mM dithiothreitol and 0.5 mg mL^−1^ BSA). The bound proteins were eluted with 1 mg/mL HA peptide (GenScript, Piscataway, NJ, USA), added with SDS sample buffer, and heat-treated at 95 ℃ for 5 min. The samples were then used in electrophoresis and western blot analysis. HA-ARF6 and ubiquitinated ARF6 were detected using anti-HA (Santa Cruz no. sc-7392) and anti-Ub (Cell Signaling Technology no. P4D1, Danvers, MA, USA) antibodies respectively, and a horseradish peroxidase-conjugated goal anti-mouse antibody (Bio-Rad). The signal was visualized with ECL Plus reagent (Cytiva-formerly GE Life Sciences, Mississauga, ON, Canada) according to the manufacturer’s instructions.

### 4.5. RNA Isolation and PCR Analysis

Total RNA samples were isolated from seedlings using the Invitrogen TRIzol reagent (Fisher Scientific) following the manufacturer’s instructions. The RNA concentration was measured with a NanoVue Plus Spectrophotometer (Cytiva-formerly GE Life Sciences, Marlborough, MA, USA) following the manufacturer’s instructions. cDNAs were synthesized using the Takara PrimeScript RT reagent Kit with gDNA Eraser and 1.0 μg total RNA in 20 μL following the manufacturer’s instructions.

For RT-PCR, the *HA-ARF* cDNA was diluted by one-third with sterile water and 1.0 μL diluted cDNA was used in 20 μL PCR reaction with 250 μM each of the primers. The PCR conditions were: 95 °C for 1 min, then 34 cycles of 94 °C for 50 s, 55 °C for 50 s, and 72 °C for 1 min, and a final extension 72 °C for 1 min. *HA-ARF6* was amplified using a primer specific to the HA-sequence (5′-CCCATACGATGTTCCAGAC) and reverse primer specific to *ARF6* (5′-TATCATGCAGATCCCTCGC). The control *At4g33380* was amplified with the forward (5′-ATGAGAAGCTGGAGGAAGC) and reverse (5′-TCAAGCCGTTACAACACC) primers. For qPCR, the reactions were performed in 20 μL, using 10 μL 2× iQ SYBR Green Supermix (Bio-Rad), 1.0 uL cDNA and 200 μM each of the primers, and run in the Bio-Rad CFX96 Real-Time PCR System. The threshold cycle (Ct) values were generated with the Bio-Rad CFX Manager Software (Version 3.0).

## Supplementary Materials

The following are available online at https://www.mdpi.com/1422-0067/21/24/9437/s1.
