# Supplementary material for: Protein Levels of Several Arabidopsis Auxin Response Factors Are Regulated by Multiple Factors and ABA Promotes ARF6 Protein Ubiquitination
Source: Int J Mol Sci. 2020 Dec 11;21(24):9437. doi: 10.3390/ijms21249437 (PMC7763875; doi:10.3390/ijms21249437)
Supplement: Supplementary file 1 [file ijms-21-09437-s001.zip › Li et al_Supplementary Materials_Final/Li et al_Supplemental Materials.pdf]

Supplementary Materials

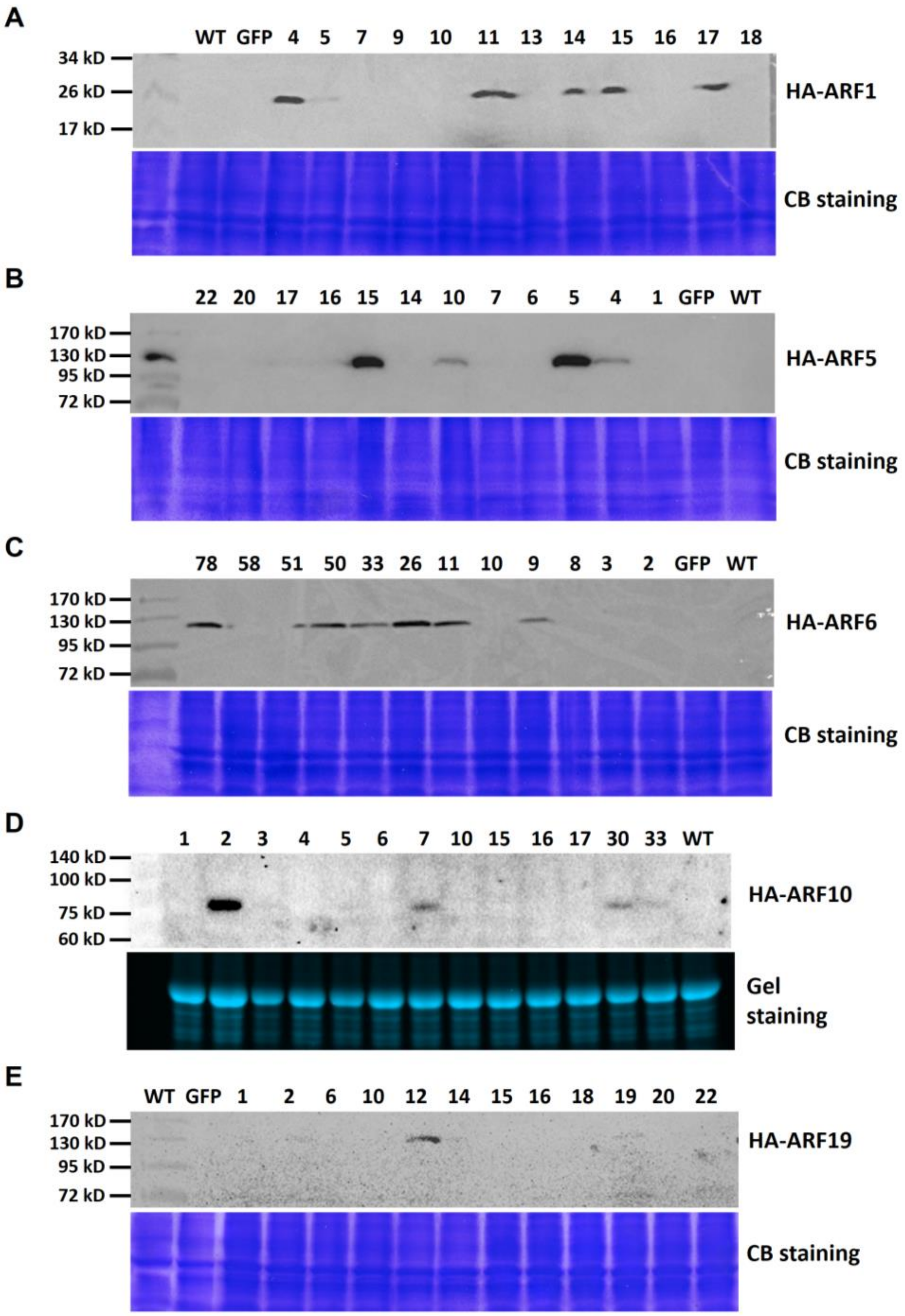

Figure S1. Survey of HA-ARF protein expression in T1 Arabidopsis transgenic lines.
Seeds were harvested from primary Arabidopsis transformants carrying an *HA-ARF* construct. The T1 seedlings (10-day old) grown in Petri plates, and about 25 seedlings from one plate were used to prepare one protein sample, representing the average expression level of the specific T1 line. For each HA-ARF construct, 12 to 13 independent T1 lines were analyzed. The protein samples (about 60 μg per lane) were used in electrophoresis and western blotting. In each subfigure, the top panel shows the western blot with an anti-HA antibody and the protein molecular weight markers are indicated at the right. To show the amount of protein, the bottom panel presents the gel stained with Coomassie Blue, except for (**D**) in which the Bio-Rad TGX gel image is shown.

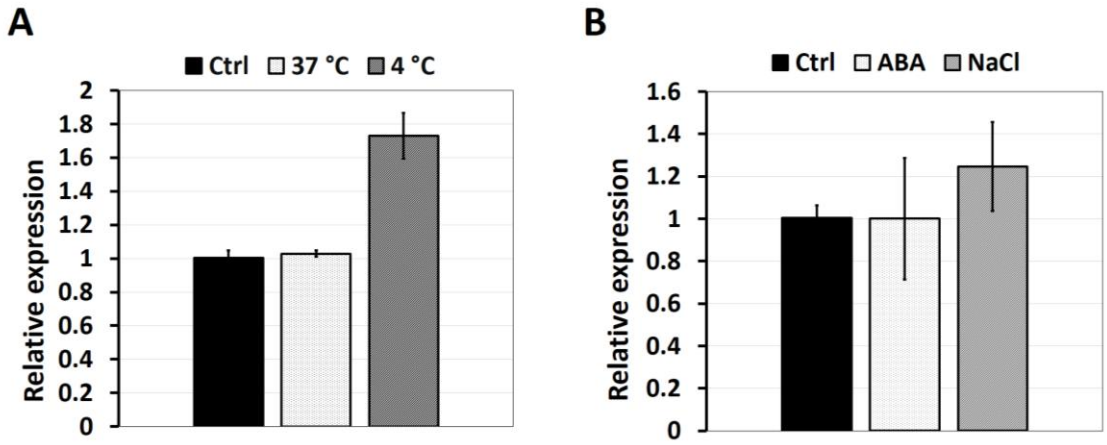

Figure S2. The relative expression level of *HA-ARF6* in Arabidopsis seedlings under several treatments determined by qPCR. Total RNA samples were isolated from treated and control seedlings and cDNAs were synthesized. qPCRs were performed in 20 μL using 10 mL 2X iQ SYBR Green Supermix, 1.0 uL cDNA and 200 mM each of the primers, and run in a Bio-Rad real-time PCR system. The Ct values are shown in Table S1. The Ct values for *At4g33380* are used to normalize and obtain the relative expression level of *HA-ARF6*. (**A**) Temperature treatments with 22 °C as the control. The data are based on four biological replicates with four technical repeats for each biological sample. (**B**) ABA (50 μM) and NaCl (120 mM) treatments. The data are based on three biological replicates with four technical repeats for each biological sample. The error bars are standard errors.

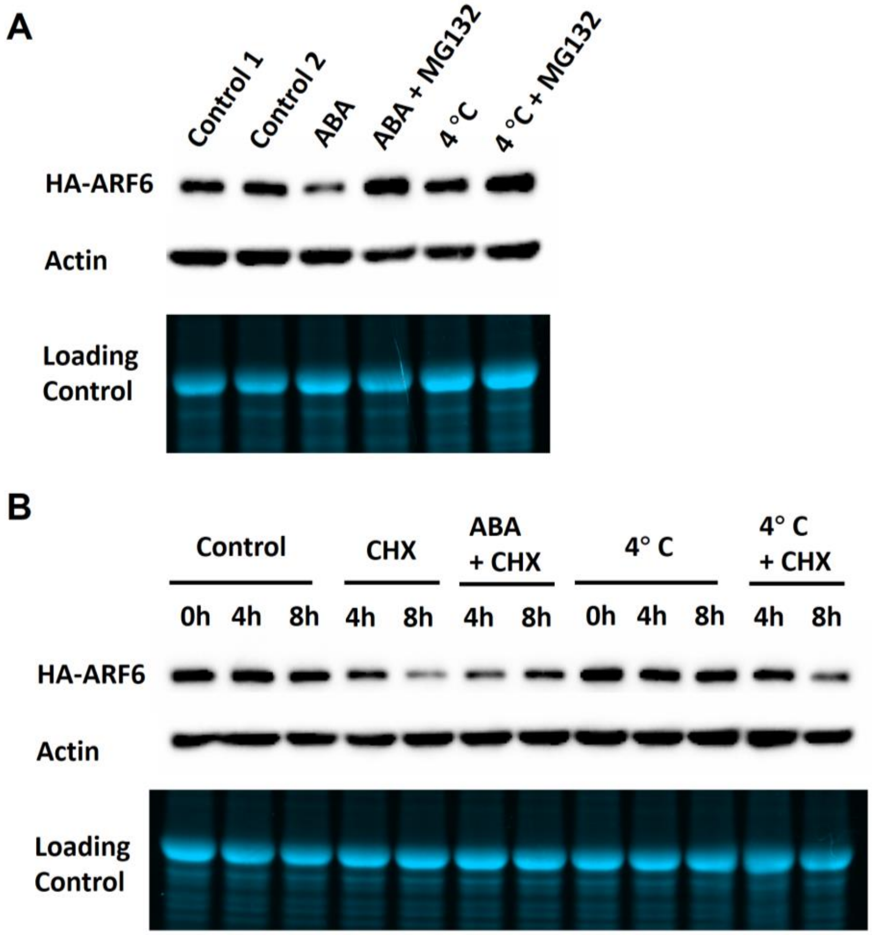

Figure S3. Effects of ABA and 4 °C treatments in the presence of MG132 or cycloheximide (CHX) on HA-ARF6 expression, using a different set of samples from the ones used in Figure 6. Transgenic *HA-ARF*6 seedlings were grown vertically in Petri plates. Seedlings (10-day old) were transferred to new plates containing a filter paper and 5 mL ½-strength MS with or without the treatment reagent. Each plate had about 25 seedlings. After the treatment, proteins were extracted and used in electrophoresis in Bio-Rad TGX mini-gel and western blotting. In each subfigure, the first row shows the western blot with an anti-HA antibody, the second row shows the western blot with an anti-actin antibody, and the third row shows the gel image. The treatment conditions are indicated above the lanes. (**A**) ABA and 4 °C treatments (8 h) with or without MG132. Control 1: normal ½ MS medium; Control 2: ½ MS medium plus 0.1% ethanol and 0.1% DMSO (ABA and MG132 stocks were prepared in ethanol and DMSO respectively); ABA: 50 μM; MG132: 25 μM. (**B**) ABA and 4 °C treatments for indicated hours with or without CHX. Control: ½ MS medium plus 0.1% ethanol and 0.1% DMSO; ABA: 50 μM; CHX: 200 μM.

Table S1. Ct values from qPCR analysis of *HA-ARF6* and *At4g33380*

Please see separate Excel file for Table S1.

Table S2. List of *ARF* primers

**Primer**	**Primer sequence**
ARF6, forward	GTCAGTCGACAATGAGATTATCTTCAGCTGG
ARF6, reverse	GTCAGCGGCCGCTTCTAGTAGTTGAATGAAC
ARF1, forward	CTGAGTCGACA ATGGCAGCTT CCAATCATTC
ARF1, reverse	CTGAGCGGCCGC TCATCTTGATCCCGCCATAG
ARF5, forward	GTCAGTCGACA ATGATGGCTT CATTGTCTTG
ARF5, reverse	GTCAGCGGCCGC TTATGAAACAGAAGTCTTAAG
ARF10, forward	CAGTGTCGACA ATGGAGCAAG AGAAAAGCTT
ARF10, reverse	CATGCGGCCGC TCAAGCGAAGATGCTGAGC
ARF19, forward	CTGAGTCGACAATGAAAGCTC CATCAAATGG
ARF19, reverse	CTGAGCGGCCGCTATCTGTTGAAAGAAGCTG
